# Galactomannan and 1,3-β-d-Glucan Testing for the Diagnosis of Invasive Aspergillosis

**DOI:** 10.3390/jof2030022

**Published:** 2016-07-04

**Authors:** Frédéric Lamoth

**Affiliations:** Infectious Diseases Service, Department of Medicine, and Institute of Microbiology, Lausanne University Hospital, Lausanne 1011, Switzerland; frederic.lamoth@chuv.ch; Tel: +41-21-314-1111

**Keywords:** *Aspergillus fumigatus*, invasive aspergillosis, fungal biomarkers, galactomannan, 1,3-β-d-glucan

## Abstract

Invasive aspergillosis (IA) is a severe complication among hematopoietic stem cell transplant recipients or patients with hematological malignancies and neutropenia following anti-cancer therapy. Moreover, IA is increasingly observed in other populations, such as solid-organ transplant recipients, patients with solid tumors or auto-immune diseases, and among intensive care unit patients. Frequent delay in diagnosis is associated with high mortality rates. Cultures from clinical specimens remain sterile in many cases and the diagnosis of IA often only relies on non-specific radiological signs in the presence of host risk factors. Tests for detection of galactomannan- (GM) and 1,3-β-d-glucan (BDG) are useful adjunctive tools for the early diagnosis of IA and may have a role in monitoring response to therapy. However, the sensitivity and specificity of these fungal biomarkers are not optimal and variations between patient populations are observed. This review discusses the role and interpretation of GM and BDG testing for the diagnosis of IA in different clinical samples (serum, bronchoalveolar lavage fluid, cerebrospinal fluid) and different groups of patients (onco-hematological patients, solid-organ transplant recipients, other patients at risk of IA).

## 1. Introduction

Invasive aspergillosis (IA) represents a major threat in patients with depressed immune system. Patients with hematologic malignancies and chemotherapy-induced neutropenia or hematopoietic stem cell transplantation (HSCT) are at highest risk [1]. However, IA is increasingly observed in patients populations with diverse types of underlying diseases and level of immunosuppression, such as solid-organ transplant recipients, patients with solid tumors, auto-immune disorders, congenital immunodeficiency or chronic pulmonary diseases [2,3]. The pathogenesis and clinical presentation of IA may differ in these settings with various degrees of invasiveness.

Diagnosis of IA remains difficult because of the lack of sensitivity of conventional culture methods. Therefore, non culture-based methods, such as detection of DNA by PCR or measurements of fungal biomarkers in blood or respiratory samples, are important adjunctive tools. The galactomannan (GM) and 1,3-β-d-glucan (BDG) assays have been included as microbiological criteria in the definitions of invasive fungal infections by the European Organization for Research and Treatment of Cancer (EORTC) and the Mycoses Study Group (MSG) [4]. However, the role and interpretation of these tests for the diagnosis of IA in various populations and clinical settings is still debated because of their limited sensitivity and specificity.

The objective of this review is to provide an overview of the current evidence of the performance of these tests and to discuss their role and interpretation in diverse patients populations.

## 2. The Tests

Characteristics of GM and BDG assays are summarized in Table 1. The test currently used for GM detection is the Platelia *Aspergillus* EIA (Bio-Rad Laboratories, Marne-La-Coquette, France). This enzyme immunoassay uses the rat monoclonal antibody EBA-2 directed against *Aspergillus* galactomannan. The antigen is first bound in the wells of the microplate coated with the EBA-2 antibody and then revealed by binding to the peroxidase-linked EBA-2 antibody resulting in a colorimetric reaction after addition of the substrate. The optical density (OD) is measured and expressed as a ratio of a control sample. Albeit specific for the detection of *Aspergillus* spp., the test may exhibit cross-reactions with other fungi, in particular those of the family *Trichocomaceae* (e.g., *Penicillium* spp., *Paecilomyces* spp.), *Fusarium* spp. and *Histoplasma capsulatum*. The Platelia *Aspergillus* EIA is validated for use in both serum and bronchoalveolar lavage (BAL) fluid samples. Testing in cerebrospinal fluid (CSF) may be considered for the diagnosis of cerebral aspergillosis on the basis of experts opinions [4,5]. The cut-off of positivity was initially set up at an OD of 1.5 and subsequently lowered at 0.5 by the manufacturer. The EORTC-MSG panel included a positive GM value as a microbiological criterion of invasive fungal infection (IFI), without specifying a threshold to define a positive result [4]. The European Conference on Infections in Leukemia (ECIL) laboratory working group recommended to consider a single value ≥0.7 or two consecutive values ≥0.5 in serum as the cut-off that should prompt further diagnostic work-up (e.g., CT-scan) for presumptive IFI [5]. Higher cut-off (single value ≥0.8 or 1.0) should be considered for BAL and CSF samples.

The diverse BDG tests detect 1,3-β-d-glucan, which is the major cell wall component of most fungal species, with the exception of fungi of the subdivision Mucoromycotina, *Cryptococcus* spp. and some other Basidiomycota (e.g., *Malassezia* spp.) that contain less 1,3-β-d-glucan in their cell wall and are usually not detected by these tests. Thus, BDG assay is not specific for the diagnosis of invasive aspergillosis and is also used for the diagnosis of invasive candidiasis. 1,3-β-d-glucan is a trigger of innate immunity and activates the coagulation cascade in horseshoe crabs. The amoebocyte lysate of two horseshoe crab species, *Limulus polyphemus* and *Tachypleus tridentatus*, are used for the Fungitell assay (Associates of Cape Cod Inc., East Falmouth, MA, USA) and the Japanese kits (Fungitec-G, Seikagaku Corporation; Wako Pure Chemicals Industries Ltd.; Maruha-Nichiro Foods Inc.; Tokyo, Japan), respectively. Upon exposure to 1,3-β-d-glucan, factor G is activated and converts the proclotting enzyme into active clotting enzyme. Colorimetric methods (Fungitell, Fungitec-G, Maruha test) or turbidimetric methods (Wako test) are used for detection of a substrate of this enzyme. These different approaches result in different cut-offs of positivity according to the test in use. The Fungitell assay is FDA approved and widely used in USA and Europe. The cut-off recommended by the manufacturer is ≥80 pg/mL for a positive test and <60 pg/mL for a negative test with an intermediate “grey zone” between 60 and 80 pg/mL. Lower cut-offs (11–20 pg/mL) are recommended for the Japanese kits. The EORTC-MSG panel has included a positive BDG test as a microbiological criterion of IFI without recommendations regarding the cut-off or the type of test [4]. New revisions of the EORTC-MSG definitions are ongoing to address these important questions.

## 3. The Performance

The performance of GM and BDG testing in serum for the diagnosis of IA has been evaluated in multiple studies and summarized in several meta-analyses [6,7,8,9,10,11]. Important heterogeneity of results has been observed, depending on the study design (cohort vs. case-control studies), the type of patients populations (onco-hematological vs. other), the method of screening (monitoring or punctual testing), the criteria used to define a positive test (cut-off of positivity, number of positive tests), and the definition of IA (use of EORTC-MSG definitions and inclusion or not of possible IA cases). 

The major conclusions of these analyses can be summarized as follows:

(1) Among high-risk patients with hematological malignancies and chemotherapy-induced neutropenia or allogeneic HSCT, both tests have a similar performance with a limited sensitivity (60%–80%) and a specificity ≥90%.
(2) The requirement of two consecutive positive tests results in a high specificity (95%–99%), with a slight loss of sensitivity.
(3) Insufficient data are available to assess the performance of these tests in solid-organ transplant recipients and other populations of immunocompromised patients at low or moderate risk of IA. However, sensitivity appears to be markedly decreased in this setting (40% or less), which can be explained by the limited angio-invasion in patients with better immune defenses compared to those with neutropenia. Concerns about poor specificity have also been raised, in particular for BDG testing in lung transplant recipients in one study [12].
(4) Data among pediatric populations are also lacking. However, the performance of these tests (especially for GM) seems to be similar to that in adults, but lower specificity has been reported in some studies [11,13,14].

The performance of GM in BAL has been assessed in several studies and two meta-analyses suggest an overall performance of 85% sensitivity and 90%–95% specificity [15,16]. The requirement of a higher cut-off (OD 1.0 versus 0.5) was associated with a substantial gain in specificity (95% vs. 90%, respectively), without loss of sensitivity. Good diagnostic accuracy of GM in BAL was also reported in solid-organ transplant recipients (in particular lung transplant recipients) with sensitivity and specificity comparable to those observed in hematologic cancer patients [17,18]. Combination of GM testing with molecular methods (PCR targeting *A. fumigatus* or *Aspergillus* spp.) has been suggested for improved performance (97% sensitivity and specificity for either GM or PCR positive) [19]. There are no data supporting the use of BDG testing in BAL.

Both the EORTC-MSG and ECIL panels have validated GM testing in CSF for the diagnosis of cerebral aspergillosis [4,5], which is supported by a recent study showing a sensitivity of 88% and a specificity of 96% in this setting [20].

Multiple possible causes of false positive results have been evoked, such as concomitant bacterial infections, β-lactam antibiotics, blood transfusions, blood-derived products, gluconate sodium-containing products (Plasma-Lyte solution, food), and renal replacement therapy [21]. For GM, the major concern was about the concomitant administration of piperacillin-tazobactam (a standard antibacterial therapy of febrile neutropenia) [22]. However, new formulations of the drug have been developed with a supposed lower risk of false positive reactivity [23,24]. For BDG, the most important cause of false positivity that was demonstrated is concomitant continuous renal replacement therapy [12]. Cross-reactivity with other fungi may also contribute to false positive results. Another important factor to consider is the high risk of contamination during the pre-analytical phase. It is recommended to test a second aliquot of the same sample to confirm a positive GM or BDG result.

Regarding false negative results, the major factor seems to be directly linked to the pathogenesis of IA with different degrees of angio-invasion and dissemination according to the level of immunosuppression of the host, with a better sensitivity of both tests in onco-hematological and/or neutropenic patients compared to solid-organ transplant recipients or other patients with less severe immunosuppression. There is no strong evidence about the role of antifungal prophylaxis in false negative results of GM or BDG.

The timing of GM or BDG positivity in serum in relationship with the appearance of the first clinical or radiological signs of IA has been investigated in some studies with controversial results depending on the screening strategy and diagnostic approach in use [25,26,27].

A robust set of data, including one meta-analysis, also supports the role of GM measurement in serum in follow-up as a marker of response to therapy and predictor of IA outcomes [28,29,30,31]. A decline of GM values within the first one or two weeks after initiation of antifungal therapy was associated with better prognosis compared to persistently high values, and we may recommend GM testing in follow-up at day 7 and 14 on the basis of these results [28,29]. However, BDG testing did not demonstrate a utility in follow-up with frequent persistence of high serum levels independently of IA outcomes [32].

## 4. The Clinical Utility

The diagnosis of IA relies on a constellation of clinical and paraclinical parameters, most of which are only presumptive and do not constitute per se a proof of infection. Because of their limited sensitivity and specificity, GM and BDG results should only be interpreted in conjunction with other clinical, radiological and microbiological criteria of IA as defined by the EORTC-MSG consensus [4]. From this point of view, two important questions should come to mind regarding the utility of GM and BDG testing in clinical practice. First, which patient should be tested? Second, when and how the test should be performed (in other words, what should be the approach or strategy of screening)?

### 4.1. Which Patient Should Be Tested?

For a given sensitivity and specificity, the positive and negative predictive values (PPV and NPV, respectively) will depend on the prevalence of the disease in a defined population and/or the pre-test probability of the disease in an individual. For instance, if we consider a prevalence of IA of about 10% in a high-risk population of patients with hematologic malignancies and chemotherapy-induced neutropenia and a sensitivity and specificity of GM or BDG around 80% and 90% respectively, the PPV would be about 50% and NPV 97%. If the patient has concomitant radiological signs consistent with IA (high pre-test probability), a positive result would definitely indicate an at least probable IA. However, assuming a similar specificity in a population with a lower IA incidence (e.g., 2%–3%), such as solid-organ transplant recipients, the PPV would fall below 20% and a positive result would likely represent a false positive result if clinical suspicion is low or moderate. Moreover, because the sensitivity is also decreased in this later population (as mentioned above), we cannot rely on a negative result to exclude the disease if clinical/radiological criteria are present.

Based on these considerations, we can conclude that GM and BDG testing has a role for routine IA diagnosis in patients with hematological malignancies during the high risk period (i.e., neutropenia or post allogeneic HSCT). However, their value is limited in other settings, such as solid-organ transplant recipients or non-neutropenic patients, in which IA prevalence is lower and sensitivity/specificity of the tests is decreased. Use of GM or BDG testing in these populations should be determined on the basis of individual assessment of the pre-test probability of IA according to host factors and clinical signs.

### 4.2. When and How the Test Should Be Performed?

We can distinguish two strategies of GM or BDG testing in serum. Punctual testing can be performed in case of clinical suspicion of IA (e.g., abnormal radiological finding consistent with IA) to precise the diagnosis. Alternatively, monitoring of BDG or GM can be performed in a selected population of high-risk patients (e.g., two or three times per week among onco-hematological patients during the neutropenic phase) in the absence of clinical signs or symptoms for early detection of IA. In this later scenario, a positive GM or BDG testing would be the first hint of the disease and trigger further diagnostic work-up including CT-scan and bronchoscopy. While monitoring strategies are applied in many onco-hematology wards, few studies have assessed the real benefit of this pre-emptive approach [33,34,35]. Thus, we cannot conclude that one algorithm is better than another. However, a monitoring strategy may be justified in high-risk patients.

Another important question is the number of positive tests required to define a positive result (i.e., one single versus two consecutive positive tests). As mentioned earlier, false positive results are relatively frequent and any positive result should, at least, be confirmed on a second aliquot of the same sample. In addition, most experts recommend the repetition of the test on a subsequent serum sample, as most studies addressing this question suggest a significant increase of specificity (from 80%–90% to ≥97%) with the requirement of two consecutive positive tests in serum for the diagnosis of proven/probable IA [7,36].

GM testing can also be performed in samples other than serum (BAL, CSF). The sensitivity and specificity of the test in BAL are higher than those reported in serum and we recommend testing of GM in BAL in all immunocompromised patients undergoing bronchoscopy for suspicion of pulmonary IA on the basis of CT imaging. The cut-off is still debated, but an OD result <0.5 virtually rules out the diagnosis, while a value ≥3 has near 100% positive predictive value [37]. While cerebral aspergillosis is a rare disease, GM testing in CSF appears as a promising diagnostic tool and can be recommended in this setting.

GM is also useful in the follow-up for assessment of therapeutic response. While radiological signs of improvement are often delayed and not evident before two weeks or more, a decline or increase of GM values in serum may be the first indicator of therapeutic success or failure, respectively. We recommend weekly monitoring of GM values during antifungal therapy of IA until values <0.5 are achieved.

## 5. Conclusions

Because microbiological documentation of IA is difficult to obtain, fungal biomarkers, such as GM and BDG, are increasingly used for the presumptive diagnosis of IA. Both tests achieve detection of IA with a comparable performance, but sensitivity and specificity are not optimal. Before requesting GM or BDG testing, the physician should ask himself the following questions:

(1) What is the pre-test probability of IA based on the prevalence of IA in this population and the clinical signs/symptoms of this patient?

(2) What are the positive and negative predictive values of the test in this particular case? (In other words, how would the result impact the decision to treat or not to treat?)

**Figure 1:**
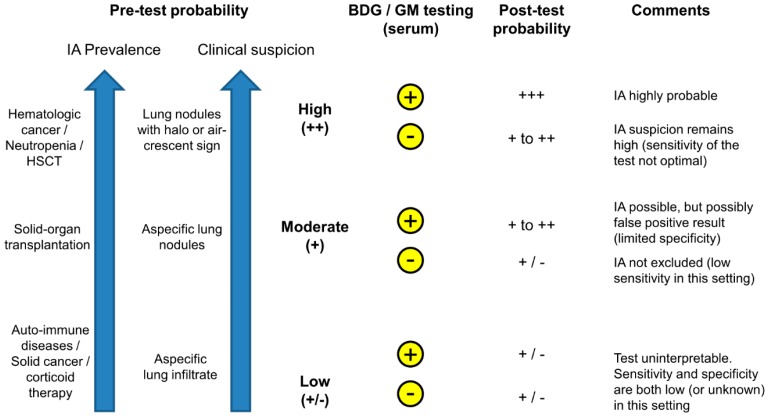
Proposed algorithm for the use of galactomannan (GM) and 1,3-β-d-glucan (BDG) testing for the diagnosis of invasive aspergillosis (IA) in clinical practice. Based on the following parameters: (1) prevalence of IA in a given population and (2) assessment of the clinical signs and symptoms of IA in a given patient, the clinician may establish the individual pre-test probability on a scale from low to high. While testing of GM and/or BDG may be useful in cases with moderate-high pre-test probability (by changing the post-test probability), interpretation of a positive or negative test is often inconclusive in low risk settings. HSCT: hematopoietic stem cell transplantation. Yellow circles: + positive test, - negative test.

In high risk patients with clinical/radiological signs of possible IA (high pre-test probability), a positive GM or BDG would probably have little impact on therapeutic decisions as these patients would be treated anyway (Figure 1). However, it may reinforce the diagnosis of IA (from possible to probable), help in differentiating IA from other mycoses, and serve as a baseline for follow-up. In these high-risk patients, GM or BDG monitoring (once or twice weekly) may also be helpful for the early detection of IA in pre-emptive strategies.

Inversely, in patients with low pre-test probability, GM or BDG results may be difficult to interpret and turn to be more confusing than helpful because of low positive predictive value (high risk of false positive results) and decreased sensitivity in non-hematological populations (Figure 1).

There is a large “in-between” or grey zone, in which GM or BDG testing may be helpful for therapeutic decisions when interpreted in conjunction with host factors and clinical/radiological findings on an individual basis.

Finally, it should be reminded that neither GM nor BDG are appropriate to detect mucormycosis, the second most frequent invasive mold infection that affects the same patients’ population as IA and has a similar clinical presentation.

In conclusion, GM or BDG testing should be targeted on the right population and right situation to offer the optimal benefit in the approach of IA diagnosis and management.

## Figures and Tables

**Table 1:** Characteristics of the galactomannan and 1,3-β-d-glucan assays.

Fungal Biomarker	Assay	Indication	Clinical Sample	Recommended Cut-off ^3^	Sensitivity/Specificity ^6^
Galactomannan	Platelia *Aspergillus* EIA (Bio-Rad Laboratories, Marne-La-Coquette, France)	Invasive aspergillosis	Serum BAL CSF	OD 0.5 OD 0.5–1 ^4^ OD 0.5–2 ^4^	60%–80%/80%–95% 85%–90%/90%–95% 85%–90%/95%–100%
1,3-β-d-glucan	Fungitell (Associates of Cape Cod Inc. (East Falmouth, MA, USA)) ^1^	Invasive aspergillosis Invasive candidiasis Other invasive mycoses ^2^	Serum	60–80 pg/mL ^5^	60%–80%/80%–95%

BAL: bronchoalveolar lavage fluid, CSF: cerebrospinal fluid; ^1^ Other assays have been commercialized in Japan: Fungitec-G (Seikagaku Corporation, Tokyo, Japan), Wako pure chemical industries Ltd (Tokyo, Japan), Maruha-Nichiro foods Inc. (Tokyo, Japan); ^2^ With the exception of mucormycosis and cryptococcosis; ^3^ Based on the cut-off of the manufacturer and experts’ recommendations; ^4^ The cut-off recommended by the manufacturer is an OD 0.5. Experts recommend to consider a higher value for BAL and CSF; ^5^ For the Fungitell assay, the manufacturer defines values <60 pg/mL and ≥80 pg/mL as negative and positive results, respectively. Values between 60 an 80 pg/mL are classified as indeterminate. Cut-off of the Japanese tests are different (Fungitec-G: 20 pg/mL, Wako and Maruha tests: 11 pg/mL); ^6^ These ranges of values are indicative and based on the results of different meta-analyses. These values may differ according to the patients’ population.
